# The amygdala via the paraventricular nucleus regulates asthma attack in rats

**DOI:** 10.1111/cns.13293

**Published:** 2020-02-03

**Authors:** Zhe Chen, Ni‐na Liu, Jian Xiao, Yue‐han Wang, Rong Dong

**Affiliations:** ^1^ Affiliated Kunshan Hospital of Jiangsu University Suzhou China; ^2^ Department of Physiology Medical School of Southeast University Nanjing China

**Keywords:** amygdala, asthma, oxytocin, paraventricular nucleus

## Abstract

**Aims:**

This study aimed to investigate the functions of the amygdala in rat asthma model.

**Main methods:**

Wheat germ agglutinin‐horseradish peroxidase (WGA‐HRP) was used for tracing from the paraventricular nucleus (PVN) to the amygdala, and nuclear lesions were performed to observe changes in respiratory function and airway inflammation.

**Results:**

This study showed that the extracellular neuronal discharged in the medial amygdala (MeA) and central amygdala (CeA), and the expression of Fos significantly increased in asthmatic rat compared to control group. The distribution of Fos‐ and oxytocin (OT)‐positive neurons and Fos/OT dual‐positive neurons evidently increased in the PVN. WGA‐HRP was injected into the PVN for tracing, and Fos/HRP‐dual‐positive neurons were observed to be distributed in the MeA. By using kainic acid (KA) to injure the MeA and CeA in asthmatic rats, expiratory and inspiratory times (TE/TI) and airway resistance (Raw) decreased, and minute ventilation volume (MVV) and dynamic pulmonary compliance (Cdyn) increased accordingly. In the bronchoalveolar lavage fluid (BALF), the number of eosinophils and the concentration of IL‐4 were lower than those of the control group, and the ratio of Th1/Th2 cells was higher than that of the control group. In the PVN, the distribution of Fos‐, OT‐positive cells and Fos/OT double‐positive cells decreased compared with those of the control group. The activities of the MeA and CeA and of OT neurons in the PVN of the rats were correlated with the occurrence of asthma.

**Conclusions:**

Asthma attack could induce neural activities in the MeA and CeA, and OT neurons in the PVN may be involved in the process of asthma attack.

## 1 INTRODUCTION

Asthma is a heterogeneous disease accompanied by chronic airway inflammation, bronchial hyperresponsiveness, and airway remodeling. The mechanism underlying asthma is complicated. A dysfunction in the ratio of Th1 and Th2 cells is the major cause of immune inflammatory diseases. The immune system does not operate independently, and its functions are regulated by the nervous and endocrine systems. The peripheral immune system can communicate with the brain via permeation through the blood‐brain barrier. The peripheral immune system can also be conducted to the peripheral organs out of the blood‐brain barrier. Signals can also be transferred to the brain via the vagal afferent nerve.^1^ The central nervous system (CNS) can activate the sympathetic nervous system, HPA axis,^2^ and vagal nerve.^3^ The signals can be transferred to peripheral organs and the nervous, endocrine, and immune systems, presenting a wide and close‐knit regulatory network. Thus, the CNS performs an important role during the asthma attack.

Previous studies showed that an increased expression of c‐fos in multiple regions of the brain in the CNS is observed when asthma occurs in sensitized animals,^4^, ^5^ indicating that the CNS may be involved in the pathogenic process of asthma attack. In the rhesus asthma model, the excitement of the nucleus of the solitary tract (NTS) is evidently improved when asthma occurs.^6^ Widdicombe has suggested that the brain's regulation of lung reflection enhances neuronal peptides in the lung, inducing neurogenic inflammation when asthma occurs.^7^ Injury in the front region of the hypothalamus can suppress the allergic and airway eosinophilic infiltration of asthma rats,^8^ indicating that the CNS may be involved in the aggravation of asthma. Therefore, to explore the involvement of the nervous system in asthma can effectively help elucidate the mechanism of asthma.

The amygdala is part of the limbic system and regulates the nervous, internal secretion, immune, and respiratory systems.^9^, ^10^ The amygdala can be activated when an individual receives immune stimulation, and electrical stimulation of the amygdala can change respiratory rhythm, frequency, and range. The amygdala is regarded as the advanced integrated center of PVN and numerous fiber projections exist between the amygdala and PVN.^11^ This study aimed to investigate the functions of the amygdala in rat asthma and their underlying mechanisms.

## 2 MATERIALS AND METHODS

### 2.1 Animals and models

Male SD rats (weighing 250‐350 g) were fed in a silent environment at 18‐25°C, housed away from strong light, and provided with rhythmic lighting in a 10 h/14 h day/night cycle and free access to food and water. The rats were fed for 1 week before the experiments were performed. All animal experiments and procedures were performed in accordance with the *Guide for the Care and Use of Laboratory Animals* published by the US National Institutes of Health**.**

According to the previous study,^12^ egg albumin (OVA, 100 mg), aluminum hydroxide (100 mg), and a mixed suspension of inactivated *Bordetella pertussis* (5 × 10^9^ copies) were intraperitoneally injected into the abdomen of the rats in the experimental group on days 1 and 3 (1 mL each time). On days 15 to 17, ultrasonic atomization was performed to transfer 1% of OVA saline solution to the rats for 20 minutes (2‐3 mL/min, particle diameter ≤5 μm). The rats with asthma presented with irritability, polypnea, forced respiration, difficulty in respiration, gasps, evident abdominal muscle shrinkage, and coughs. The injected normal saline (NS) (pH 7.2‐7.4) and OVA were maintained at 37°C.

### 2.2 Pulmonary function test

Asthmatic and normal rats were anesthetized with 0.4% pentobarbital sodium (40 mg/kg, intraperitoneal injection). The anal temperature was approximately 36‐38°C. The limbs and the head were fixated in a supine position. The trachea was separated, and an inverted “T”‐shaped incision was cut in the trachea. A pipe that was connected to the airflow exchanger was inserted. The esophagus was incised transversely; then, a water injection catheter with four‐side holes was inserted, and a venous pressure transducer was connected to measure the pressure in the esophagus to replace the intrathoracic pressure. Respiratory flow and esophageal pressure signals were collected by an RM6240 multichannel physiological signal acquisition, and processing system connected to a computer to record lung function within 30 minutes before and after the onset of asthma. The respiratory frequency (RF), tidal volume (V_T_), and minute ventilation volume (MVV) were calculated. The signal collection rates, filtering parameters, and magnification were consistent throughout the experiment.

### 2.3 Hematoxylin and eosin staining, BALF and IFN‐γ and IL‐4 detection

Asthma and control rats were anesthetized with 0.4% sodium pentobarbital solution (40 mg/kg, intraperitoneal injection). The rat chest cavity was exposed, and the right atrial appendage was incised, and the right hilar was ligated, and the right lower lung was collected for histopathological staining. Three milliliters of 0.3% phosphate buffered saline (PBS) solution at pH 7.4 and 37°C was injected into the trachea and recycled five times. BALF was collected in a centrifuge tube, incubated in a water bath at 4°C, and centrifuged at 1250 *g* at 4°C for 10 minutes. The pellets were collected for eosinophil and white blood cell counting. The supernatant was stored at −20°C, and the concentrations of IFN‐γ and IL‐4 were determined through ELISA in accordance with the manufacturer's instructions.

### 2.4 Discharge recording and histological localization of the MeA and CeA

Rats were anesthetized with 0.4% sodium pentobarbital solution (40 mg/kg, ip) and fixed on a brain stereotaxic apparatus. Spike discharge recording from left CeA or MeA neural units was performed with reference to the Paxison and Watson rat brain map (left MeA positioning, AP −2.8 mm, LR 3.3 mm; left CeA positioning: AP −2.4 mm, LR 4.0 mm). A glass microelectrode with a stepper motor (tip diameter <1 µm, filled with 0.5 M sodium acetate and 2% guanamine sky blue, with a resistance of approximately 3 kΩ) was inserted at 8.8‐9.3 mm below the surface of the brain. In this manner, the stable spontaneous discharge of MeA neurons could be recorded. Spontaneous discharge signals from stable CeA neurons could be recorded at 7.8‐8.4 mm below the brain surface. Subsequently, the tail vein was injected with OVA to perform a challenge, and the injection was completed within 1 minute. The control rats were injected with saline via the tail vein. The change in discharge was recorded continuously for 30‐40 minutes. After the recording was completed, the glass microelectrode passed a direct current of 25 μA for 20 minutes through a cathode, and microelectrophoresis was applied to stain the position with guanamine sky blue. After the test was performed, the brain was decapitated and fixed in 4% paraformaldehyde (PFA). The brain was sliced to validate the position of the electrode.

### 2.5 Fos and OT immunohistochemistry

To study the expression of Fos in the MeA, CeA, and PVN in asthmatic rats, rats were divided into an asthma group (exposed to 1% OVA for 20 minutes to induce asthma), a saline group, a normal group, and a sham group. The saline group rats were sensitized and challenged with NS instead of the OVA suspension. The normal group rats were fed in the normal environment for 48 hours, and no surgery was conducted. The sham group rats were manipulated during sensitization, stimulation, and surgery without the administration of drugs to rule out the effect of surgical operations on Fos expression.

Rats were anesthetized by intraperitoneally injecting 0.4% sodium pentobarbital (40 mg/kg) 2 hours after stimulation. Rats were transcardially perfused with PBS, followed by 4% PFA in PBS. Afterward, the brain tissue was taken, and the interosseous segment containing the amygdala and hypothalamic PVN was excised, fixed in 4% PFA for 4 hours at 4°C, and immersed in 30% sucrose solution at 4°C for 48 hours.

Frozen continuous coronal sections (thickness 30 μm) were obtained for detecting Fos via immunohistochemistry (IHC). Sections were incubated with 3% H_2_O_2_ for 15 minutes to block endogenous peroxidase activity, then washed with 0.3% PBS (3 × 5 minutes), incubated for 1 hour at room temperature with a blocking solution (10% goat serum), and subsequently incubated overnight with the primary antibody (rabbit anti‐Fos; 1:500; Santa Cruz or rabbit anti‐OT; 1:1000; Millipore). The tissue was washed with 0.3% PBS (3 × 5 minutes), followed by incubation for 1 hour at room temperature with a biotinylated secondary antibody (goat anti‐rabbit; 1:300; Abcam). After washing with 0.3% PBS (3 × 5 minutes), sections were incubated for 30 minutes with avidin/biotinylated horseradish peroxidase (HRP), washed with 0.3% PBS (3 × 5 minutes), and reacted with DAB as a chromogen. Sections were observed using an Olympus light microscope.

Oxytocin is secreted in the PVN and supraoptic nuclei (SON) of the hypothalamus and affected by circadian rhythm. To avoid the effects of circadian rhythm on the OT, all the experimental time was matched, and the animal experiments were performed from 8:00 to 12:00 am The OT density (mean of density) measured was mainly located on PVN, which OT expression was mainly distributed on. One section in every six consecutive pieces of brain sections was selected, and total eight brain sections for immunohistochemistry were selected for statistics in each experimental animal. The numbers of Fos‐positive neurons and the mean densities of OT‐positive neurons were determined using Image‐Pro Plus (IPP).

### 2.6 WGA‐HRP retrograde tracing

Asthmatic rats were anesthetized by intraperitoneally injecting 0.4% sodium pentobarbital solution (40 mg/kg) and then fixed on a stereotaxic instrument. By using the coordinates from the Paxison and Watson rat brain map (PVN: AP 1.8 mm, R 0.4 mm, H 7.4 mm), 0.05 μL of 4% WGA‐HRP was slowly injected into one side of the PVN by using a 1 μL microinjector with a microtube. The microinjector was activated at a constant rate (0.01 μL/min) for approximately 5 minutes. After the drug was injected for 20 minutes, the microinjector was quickly pulled out, the muscles and the skin were sutured, and the incised skin was disinfected. After suturing was performed, the rats were subjected to single cage feeding. After 48 hours of survival, the brain tissue containing the amygdala was collected as described above.

### 2.7 HRP and Fos double staining

The sections were subjected to WGA‐HRP color reaction via a tetramethylbenzidine (TMB) assay.^13^ For the TMB assay, the sections were collected in 0.1 M PB (pH 7.2‐7.4) and washed thrice in distilled water for 2‐3 minutes. The sections were presoaked for 20 minutes in the TMB reaction solution, and the incubation was performed in the dark (15°C–20°C). Approximately 0.7 mL of 0.3% H_2_O_2_ was added to each 100 mL of TMB solution every 10 minutes. The change in color was observed closely under a microscope and stopped with a stop solution (1:4 diluted 0.2 M PB (pH 5.0‐5.4) 3‐6 times for 2‐4 minutes). The TMB reaction product was strengthened with a DAB/CoCl_2_ strengthening solution. The degree of coloration was closely observed under the microscope, and coloration was terminated with distilled water. After full elution was achieved, a Fos immunohistochemical reaction (yellow DAB coloring agent) was conducted.

### 2.8 MeA and CeA lesions by using kainic acid (KA)

To further explore the role of amygdala in asthmatic rats, we detected changes in eosinophils, IL‐4, IFN‐γ, and Th1/Th2 in lung function and the alveolar lavage fluid after the MeA and CeA were injured. Changes in the distribution of Fos/OT double‐labeled neurons in the PVN were observed. The rats were divided into the experimental and control groups.

The experimental group was divided into asthmatic rats with medial amygdala lesion (L‐MeA) group and asthmatic rats with central amygdala lesion (L‐CeA) group.

The control groups were divided into a saline group, an asthma group, a sham medial amygdala lesion (S‐MeA) group, and a sham central amygdala lesion (S‐CeA) group. The saline group rats were sensitized and challenged with NS instead of OVA suspension.

MeA or CeA lesions are described below. Rats were anesthetized by intraperitoneally injecting 0.4% sodium pentobarbital solution (40 mg/kg) and then fixed on a stereotaxic instrument. Kainic acid (0.2 μg/0.1 μL) was injected into the left and right MeA or CeA with a micro‐syringe. The needle was left for 4 minutes to prevent the drug solution from overflowing, and the wound was sutured after the operation. The S‐MeA or S‐CeA group rats received the same surgical procedure but did not receive KA treatment.

After 7 days, the pulmonary function of the rats was measured. The BALF was subjected to eosinophil counting and cytokine detection.

### 2.9 Fos/OT immunohistochemistry in the PVN following MeA or CeA lesions

Fos immunohistochemical staining was performed, and blue DAB chromogenic reagent was used for color development. The degree of color development was closely observed under the microscope, and the color reaction was terminated in time with distilled water. The tissues were washed thoroughly with 0.01 mol/L PBS four times for 7 minutes and then fully immersed in rabbit anti‐OT for incubation. The tissues were colored with yellow DAB chromogenic reagent, and the degree of coloration was closely observed under the microscope.

### 2.10 Statistical analysis

Data were expressed as the mean ± SD and analyzed for significant differences using SPSS 17.0 software. Comparisons among multiple groups were performed using one‐way analysis of variance (ANOVA), and Student's *t* test was used for comparison of two groups. A *P*‐value <.05 was considered statistically significant.

## 3 RESULTS

### 3.1 Pulmonary function and airway inflammation in asthmatic rats

After rats received the asthma challenge, irritability, scratching, abdominal muscle contraction, nodding breathing, wheezing, cough, and other asthma symptoms occurred. The peak of the asthma symptoms was reached in 15 minutes and lasted 30 minutes. In severe cases, purpura appeared, and the limbs went soft. The rats in the control group were allowed to inhale NS under the same conditions, and no significant change in behaviors was observed.

**Figure 1:**
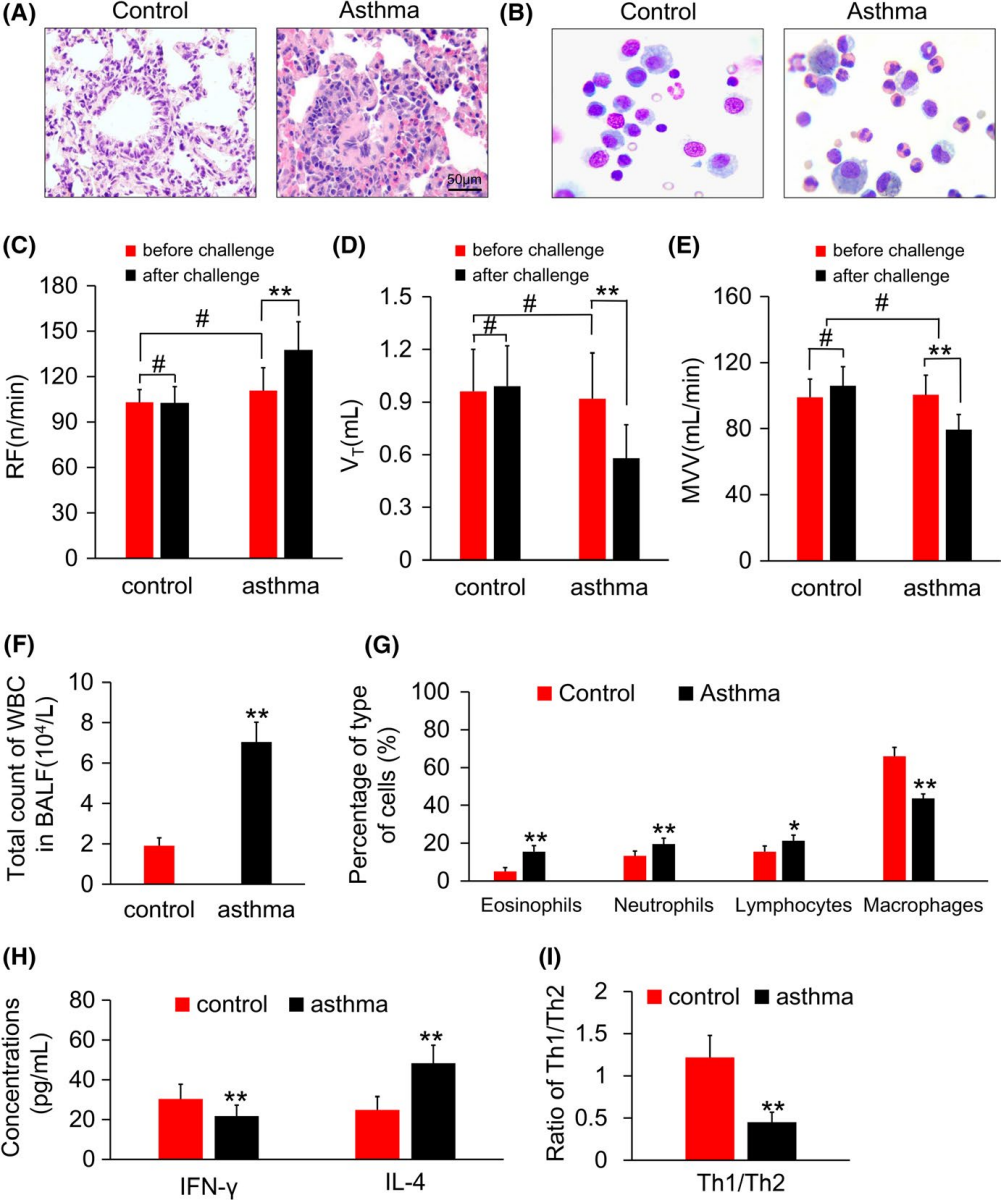
A, HE staining in the lung tissue. B, Eosinophil in BALF. C, D, E, The respiratory rate, tidal volume, and the minute ventilation volume before and after challenge. F, Cell counts in BALF. G, Percentage of cells in BALF. H, Concentrations of IFN‐γ and IL‐4. I, Ratio of Th1/Th2. BALF, bronchoalveolar lavage fluid. *P < .05, **P < .01, and #*P* > .05, respectively

A large area of inflammatory cell infiltration was observed in the bronchial wall and pulmonary interstitial space of the asthmatic rats. Eosinophils and neutrophils were the main components. The bronchial epithelium was not well arranged. Epithelial cells were exfoliated, and mucus plugs were observed in the lumen. The interstitial space was thickened. The bronchial, alveolar, and pulmonary interstitial structures of the lung tissue sections of the control group were intact, and the epithelium was arranged neatly. No epithelial cells were found in the lumen, and no mucus plug was observed. (Figure 1A).

The number of eosinophils in the BALF of the asthmatic rats was significantly higher than that of the control group (*P* < .01); the total number of cells in the BALF of the asthmatic rats was higher than that of the control rats mainly because of the increase in eosinophils and lymphocytes (*P* < .01). (Figure 1B,F,G).

After the OVA challenge, respiratory rate increased, tidal volume decreased, and ventilation per minute decreased, resulting in shallow breathing in the asthmatic rats (*P* < .01). (Figure 1C,D,E).

The concentration of IFN‐γ in the asthmatic rats was significantly lower than that in the control group (*P* < .01), and the content of IL‐4 was significantly higher than that in the control group (*P* < .01). The Th1/Th2 ratio in the BALF was significantly reduced during the asthma challenge in the affected rats compared with that of the control group (*P* < .01). (Figure 1H,I).

### 3.2 Changes in the extracellular discharge of MeA and CeA neurons in sensitized rats with asthma

**Figure 2:**
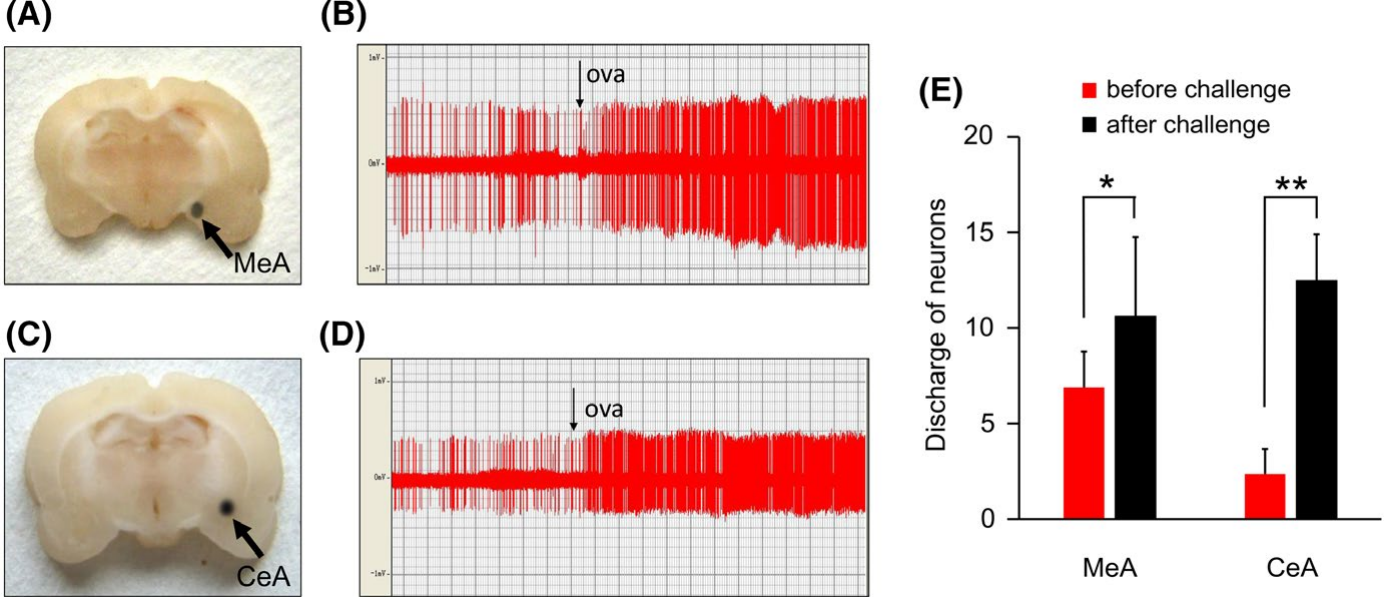
A, C, Locations of MeA and CeA. Black Arrows. B, D, E, The frequency of discharge in MeA and CeA before and after OVA challenge in asthmatic rats. *P < .05 and **P < .01, respectively

The neurons in the MeA and CeA recorded in this experiment showed excitatory enhancement after the OVA challenge in asthmatic rats (Figure 2). After the stimulation, the frequency of discharge increased from 6.89 ± 1.88 Hz to 10.63 ± 4.12 Hz (*P* < .05) in the MeA and increased from 2.37 ± 1.30 Hz to 12.47 ± 2.43 Hz (*P* < .01) in the CeA. No significant change was observed in the control rats.

### 3.3 Fos expression in the PVN, MeA, and CeA in asthmatic rats

**Figure 3:**
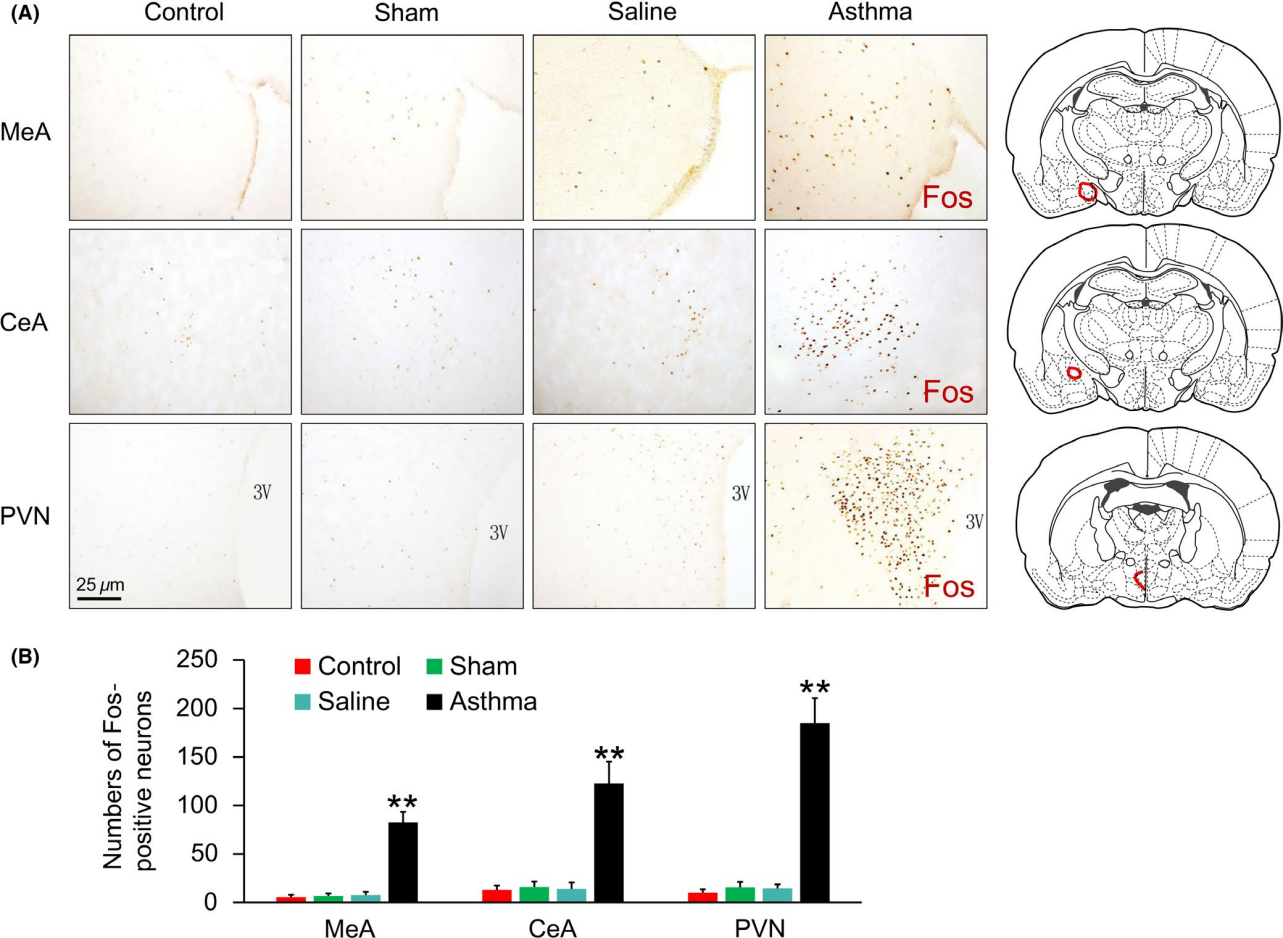
A, Fos expressions in the MeA, CeA, and PVN in the control, sham, saline, and asthma group rats. Fos‐positive neurons were brown staining. B, Fos‐positive neurons in the asthma group rats were more than that in the each other group. **P < .01

The immunohistochemical sections of rat MeA, CeA, and PVN revealed that in the asthmatic rat, the nuclei of Fos‐positive neurons were brown yellow, the cytoplasm was not stained, and the cell bodies and processes could not be seen. The Fos immunoreactive‐positive substances in the normal control, sham operation, and saline control groups showed a scattered distribution in the PVN, MeA, and CeA, no dense distribution areas, mostly light staining, small numbers, and no significant differences among the groups (Figure 3A). After asthma onset, the PVN, MeA, and CeA in the asthmatic rats were filled with Fos‐positive neurons, which showed a symmetric distribution that was statistically significant compared with the brain regions from the normal control group, sham operation, and NS control groups (*P* < .01) (Figure 3B).

### 3.4 OT‐positive neurons in the PVN of asthmatic rats

**Figure 4:**
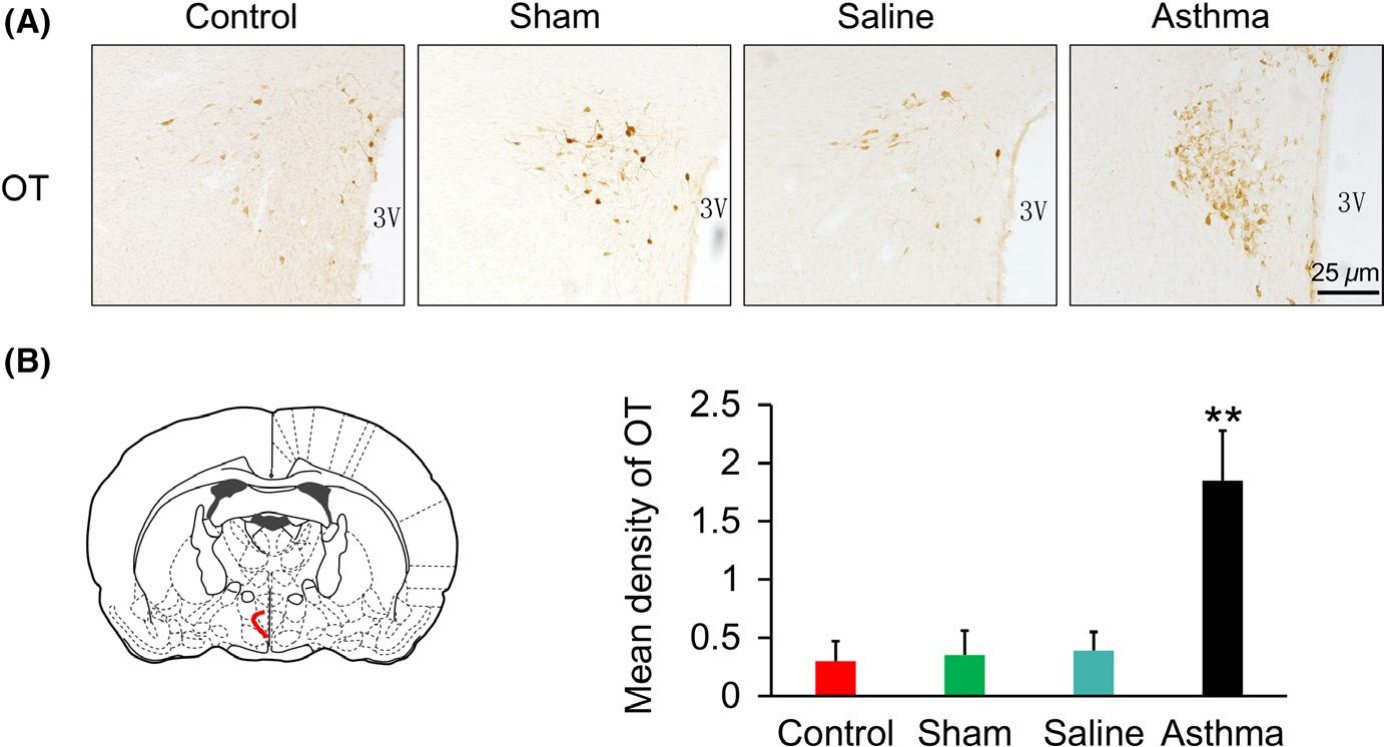
A, Oxytocin expressions in the PVN in the control, sham, saline, and asthma group rats. OT‐positive neurons were yellow staining. B, The number of OT‐positive neurons in the asthma group rats was more than that in the each other group. **P < .01

In the asthmatic rats, the cytoplasm of OT‐positive neurons was brown yellow, and the background staining was pale yellow (Figure 4). OT immunoreactive neurons in the hypothalamic slices of the rats in each control group were only scattered in the PVN. No dense distribution areas and mostly light staining were observed.

### 3.5 Distribution of WGA‐HRP‐labeled neurons in the amygdala of

**Figure 5:**
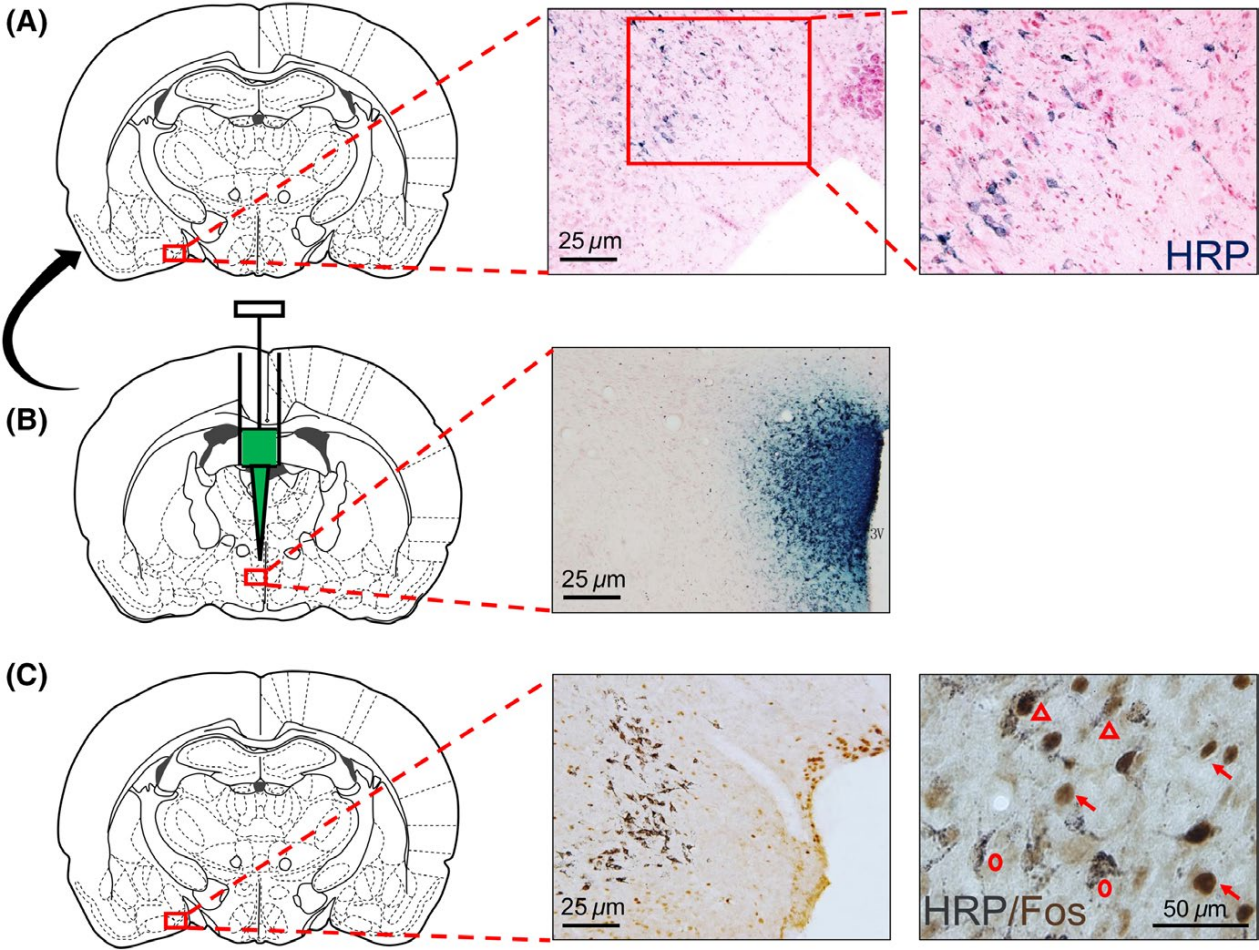
A, Horseradish peroxidase single‐labeled neurons (blue staining) were distributed in the MeA. B, WGA‐HRP microinjection zone in the PVN. C, Three kind of labeled neurons were distributed in the MeA. Fos labeled neuron was yellow staining (red arrow), and HRP‐labeled neuron was black staining (red circle), and HRP/Fos double‐labeled neuron was yellow and black staining (red triangle)

The rat amygdala is located in the anterior hippocampus of the temporal lobe and in front of the lower ventricle of the lateral ventricle. The internal structure of the amygdala is complicated, with a central subnucleus, lateral subnucleus, and basal nucleus. HRP‐labeled neurons showed blue particles in the cytoplasm, and some of them showed cell bodies and contours, but the nucleus was not colored (Figure 5A). The WGA‐HRP injection zone was confined to one side of the PVN (Figure 5B). HRP‐labeled neurons were densely distributed in the MeA (53.68 ± 5.35), and no obvious HRP‐positive neurons were found in the CeA and other subnuclei.

Positive neurons in the rat amygdala slices are categorized into three types, namely, Fos and HRP single‐labeled neurons and HRP/Fos double‐labeled neurons (Figure 5C). The HRP/Fos double‐labeled neurons were mainly found in the ventral part of the MeA. The number of HRP/Fos double‐labeled cells was 19.24 ± 0.79, accounting for 35.95% of the total number of WGA‐HRP retrograde‐labeled neurons and the total number of Fos‐positive neurons (14.02%).

### 3.6 Effects of the chemical damage in the MeA and CeA on asthmatic rats

**Figure 6:**
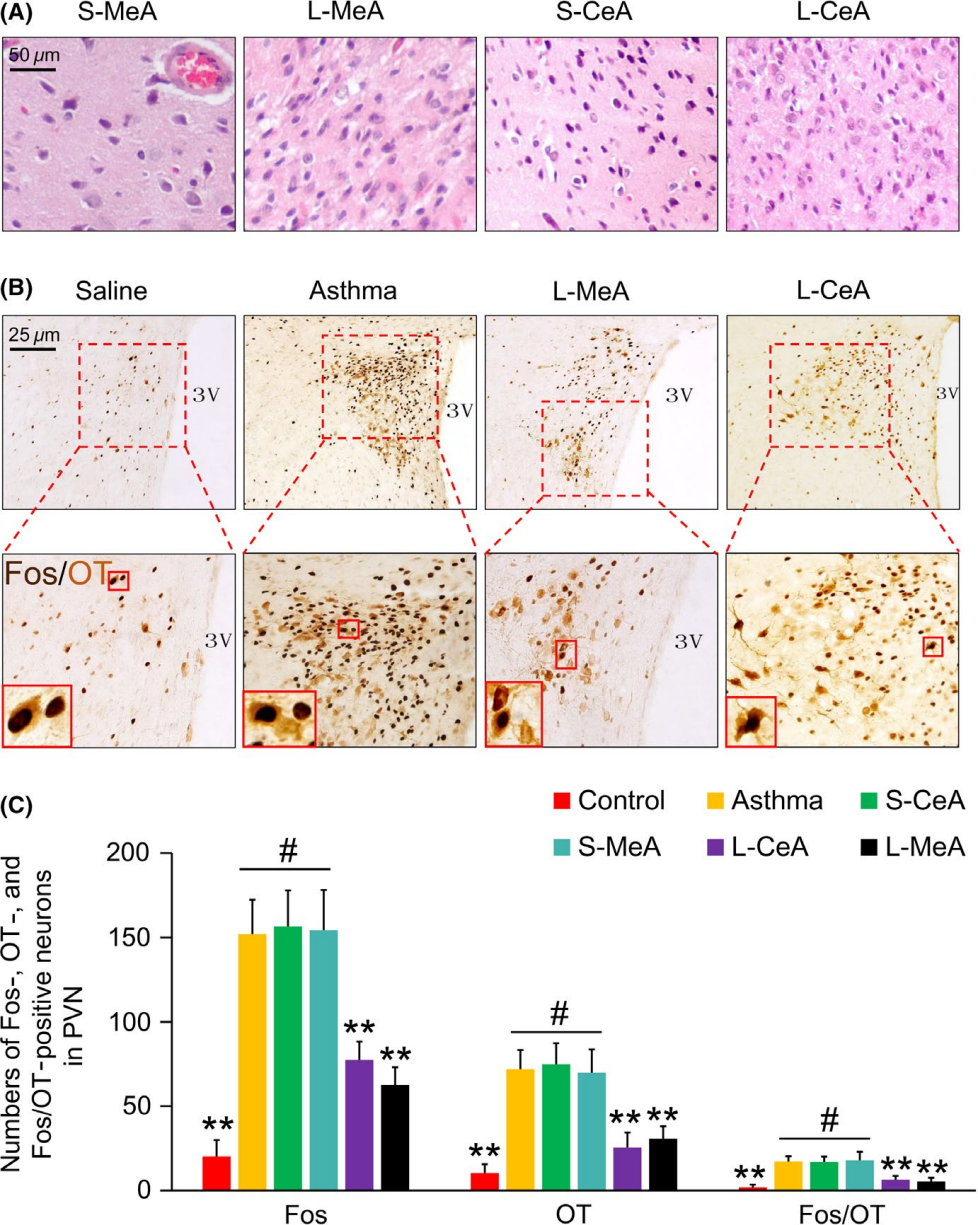
A, HE staining of MeA or CeA neurons after KA injection or sham operation. B, Fos/OT labeled neurons in the PVN in each group. Results in S‐CeA and S‐MeA were not shown. C, Fos, OT‐positive neurons, and Fos/OT double‐labeled‐positive neurons counts in each group. **P < .01 and #*P* > .05, respectively

KA is an excitatory neurotoxic drug that selectively destroys neuronal cell bodies in the injection zone. The MeA or CeA neurons in the asthmatic rats receiving the KA injection were degenerated and necrotic, the surrounding microglial cells proliferated, and the brain tissue softened compared with those in the control groups. (Figure 6A).

The number of Fos, OT‐positive neurons, and Fos/OT double‐labeled‐positive neurons in the PVN was significantly higher in the asthma group than those in the control groups (*P* < .01) but reduced after MeA or CeA lesion (*P* < .01) (Figure 6B,C). This indicates that the MeA and CeA might be involved in asthma attack through the OT neurons of the PVN.

**Figure 7:**
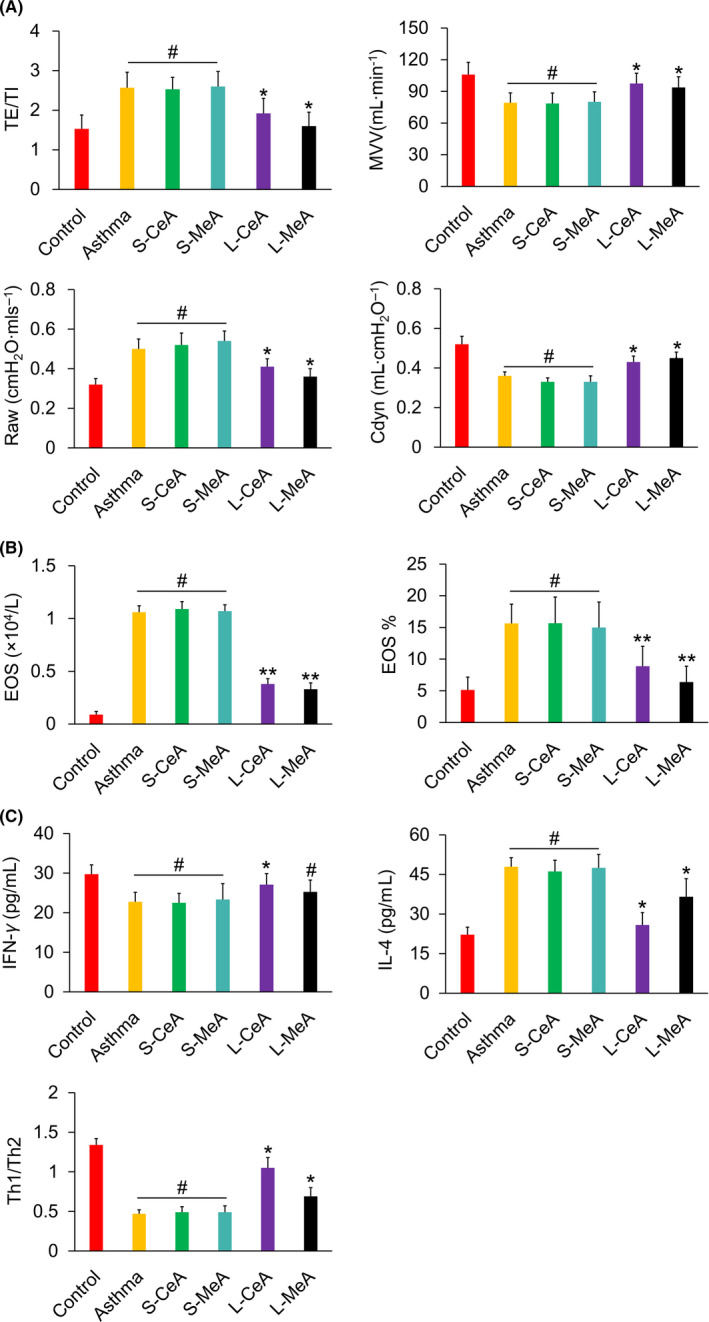
A, Changes of TE/TI, Raw, MVV, and Cdyn in each group. B, Eosinophil counts and percentage in BALF in each group. C, IFN‐γ and IL‐4 concentrations and Th1/Th2 ratio in each group. TE/TI, expiratory time course/inspiratory time course ratio. BALF, bronchoalveolar lavage fluid; Cdyn, dynamic pulmonary compliance; MVV, minute ventilation volume; Raw, airway resistance. *P < .05, **P < .01, and #*P* > .05, respectively

After MeA or CeA lesion in the asthmatic rats, the expiratory time course/inspiratory time course ratio (TE/TI) and airway resistance (Raw) decreased (*P* < .01), and minute ventilation volume (MVV) and dynamic pulmonary compliance (Cdyn) increased (*P* < .01) compared with those of the asthma group, whereas no significant change was found compared with the sham operation group, indicating that the lesion of MeA and CeA could aid in alleviating asthma symptoms. (Figure 7A).

The lesions in the MeA or CeA could affect the proportion of various cells in the BALF of the asthmatic rats; the proportion of eosinophil in the lesioned animals was significantly lower than that of the asthma group or the sham group (*P* < .01). (Figure 7B).

The lesion in the MeA showed no significant effect on the IFN‐γ content (*P* > .05), but the content of IFN‐γ increased after the CeA lesion with respect to that of the asthma group. Both lesions resulted in a decreased IL‐4 content (*P* < .01) and an increased Th1/Th2 ratio (*P* < .01). The results indicated that the MeA and CeA partially regulated the immune function of asthmatic rats, and the CeA showed a relatively strong regulatory effect on cytokines. (Figure 7C).

## 4 DISCUSSION

The amygdala is located in the hippocampus and is part of the limbic system. The structure and function of the amygdala are complicated and to date have not been clearly defined. Their structure and function are not only related to olfaction and its joint reflex but are also associated with a series of complicated reflexes and even conditioned reflexes, such as visceral activity, physical activity, endocrine activity, emotion, learning, and memory.^14^, ^15^, ^16^

The expression of Fos in the amygdala, mainly in the MeA and CeA, was increased in the asthmatic rats. Electrophysiological results also showed an increase in neuronal discharge in the MeA and CeA during asthma. Respiratory function tests confirmed expiratory dyspnea during asthma attack. The dyspnea was characterized by an increase in the respiratory rate, an increase in the TE/TI, a decrease in the tidal volume, and a decrease in the ventilations per minute. Changes in respiratory function during an asthma attack might stimulate amygdala activity. The amygdala contains respiratory‐related, chemically sensitive neurons, and amygdala cells are stimulated during respiration.^17^ Air deficiency or hypercapnia can activate the amygdala.^18^, ^19^, ^20^

An asthma attack may be to the result of decreased blood oxygen partial pressure and elevated CO_2_ partial pressure; thus, a number of mechanisms may activate the amygdala in an asthma attack, including direct action on chemically sensitive neurons in the amygdala, direct projections through the ventral region of the medulla oblongata^21^ or through the covered midbrain,^22^ and indirect projections of the hypothalamus, thalamus, and septal nucleus.^23^ After activation, the amygdala regulates the asthma attack by regulating the ventral lateral respiratory group. Our experiments showed that the MeA and CeA could affect the respiratory function of the asthmatic rats, and no significant difference was observed between the two lesion groups, suggesting possible synergistic effects between the MeA and CeA during an asthma attack. When one of the nuclei was destroyed, the direct regulation between the nuclei and respiratory neurons was also destroyed.

Neuronal discharge in the MeA and CeA was increased during the asthma attack, and the expression of Fos protein was also increased, possibly because of the regulation of immune functions. The amygdala is associated with autonomous regions, such as the NTS. The autonomic nervous system is the main source of regulation by the brain of the immune system and is involved in the feedback of immune information to the brain. Immune challenges cause increased Fos expression in the amygdala.^24^ Lesions in the amygdala can significantly attenuate the formation of a conditional immunosuppressive state,^25^ while the normal immune response is unaffected. Electrochemical damage to the central amygdala alters the cellular immune response, indicating that the nucleus is involved in neuroendocrine immune interactions.^26^

Asthma involves neuroimmune‐endocrine interactions. The results of this study confirmed that Fos‐positive neurons in the MeA and CeA increased in asthma, suggesting an increase in MeA and CeA activities during asthma. The amygdala belongs to a part of the limbic system, which is involved in neuroendocrine and neuroimmune regulation. Therefore, the concept of the limbic system‐limbic hypothalamo‐pituitary‐adrenocortical axis has been proposed. The amygdala affects PVN activity, which is known to be involved in asthma.^27^, ^28^ This study also found that the expression of Fos in PVN neurons was also enhanced in asthma. The CeA is a potential site for the regulation of the HPA axis,^29^ and studies have shown that the MeA may be directly implicated in the regulation of neurosecretion in the PVN.^11^ Both the MeA and CeA emit fibers that project to the PVN.^30^, ^31^, ^32^ HRP/Fos dual‐labeled neurons were mainly distributed in the MeA, suggesting that the MeA might regulate asthma attacks through the PVN.

The PVN is the integration and regulation center of neuroendocrine and autonomic (primarily sympathetic) functions, with neurons playing essential roles in controlling traditional autonomic functions,^33^ and has been shown to affect immune function.^34^, ^35^ PVN neurons mainly synthesize and secrete OT and vasopressin, with the number of OT neurons higher than that of vasopressin neurons.^36^ Oxytocin predominantly locates in PVN and SON. It plays a role in many important functions, such as sexual reproduction, cardiovascular, and digestive system. During stress, HPA axis can be activated, thereafter boosts the synthesization of oxytocin. Asthma is a disease of allergic airway inflammation. Stress occurs in acute asthma attack, which can activate many brain areas, thus increases the production of oxytocin. The results of this experiment confirmed that the activity of OT neurons in the PVN increased in asthmatic rats, possibly because of the stimulation of immunogenic mediators released by peripheral cells, such as eosinophil. OT levels are elevated in the immune response.^37^ Intravenous and intraventricular injection of LPS and IL‐1 can lead to an increased expression of Fos protein in OT neurons in the PVN of experimental animals.^38^ These findings indicate that OT can excite neurons in the PVN during the immune response. NTS emitting fibers to the PVN may be involved in the activation of OT neurons in the PVN. Activated OT neurons may be involved in cardiorespiratory activities regulated by regulating autonomic ganglion neurons in the brainstem.^39^

Oxytocin is not only related to immune function but is also implicated in respiratory regulation.^40^, ^41^ The OT neurons in the PVN project directly into the airway‐associated vagal preganglionic neurons^42^ and pre‐Botzinger complex (PBC), thereby stimulating the PVN, and the released OT acts on the OT of the PBC, changing the discharge activity and frequency of the diaphragm.^43^ In asthmatic rats, the expression of Fos in OT neurons in the PVN increased, indicating that OT neurons in the PVN were excited during asthma and that excited OT neurons might affect asthma attacks by directly regulating respiratory‐related neurons.

The results of this study confirmed that after performing lesions in the MeA, the activity of OT neurons in the PVN decreased, suggesting that the MeA might directly regulate the activity of OT neurons in the PVN, which in turn act on the vagus preganglionic neurons of the medulla, thereby regulating asthma. This study did not find a direct projection of the CeA to the PVN that could be responsible for regulating asthma, but the CeA exhibits a large amount of fibers projected to the bed nucleus of the stria terminalis (BNST). The BNST is generally considered to be a relay station for the amygdala and PVN and is thought to regulate PVN activity.^44^ In addition, reciprocal projection occurs between the MeA and CeA, which may interact to affect PVN neuronal activity.

## 5 CONCLUSIONS

Asthma attack could induce neural activities in the MeA and CeA, and OT neurons in the PVN may be involved in the process of asthma attack.

## CONFLICT OF INTEREST

The authors declare no conflict of interest.
